# GDF15 orchestrates mitochondrial-immune crosstalk via SMAD7-HIF-1α-PKM2 cascade to attenuate septic liver injury

**DOI:** 10.3389/fimmu.2025.1712741

**Published:** 2026-01-22

**Authors:** Xiandong Kuang, Zhili Niu, Wenqiang Liu, Zhaoyang Huang, Shuo Li, Ye Zhang, Li Wang, Xin Cai, Faxi Wang, Pingan Zhang

**Affiliations:** 1 Department of Clinical Laboratory, Institute of Translational Medicine, Renmin Hospital of Wuhan University, Wuhan, China; 2 Central Laboratory, Renmin Hospital of Wuhan University, Wuhan, China

**Keywords:** GDF15, metabolic-inflammatory dysregulation, mitochondrial dysfunction, mitochondrial resuscitation, sepsis, SMAD7-HIF-1 α axis

## Abstract

**Background:**

Sepsis-induced multi-organ failure involves pathological crosstalk between mitochondrial dysfunction and hyperinflammation, yet endogenous protective mechanisms remain incompletely defined. This study investigates Growth Differentiation Factor 15 (GDF15) as a potential regulator of sepsis tolerance.

**Methods:**

Using LPS-challenged mouse endotoxemia and a murine macrophage (RAW264.7) cell line model, we assessed GDF15’s functional role through: (1) recombinant Adeno-Associated Virus serotype 8 (rAAV8)-mediated tissue-specific overexpression, (2) siRNA knockdown, (3) pharmacological modulation (BAY 87-2243/Hypoxia-Inducible Factor 1-alpha (HIF-1α) inhibitor, Shikonin/PKM2 inhibitor, Asiaticoside/SMAD7 activator), and (4) comprehensive metabolic-inflammatory phenotyping including mitochondrial complex integrity (assessed via UQCRC1, Ubiquinol-Cytochrome c Reductase Core Protein 1), cytokine dynamics (TNF-α, IL-6) and lactate metabolism.

**Results:**

LPS challenge induced time-dependent mitochondrial dysfunction concurrent with cytokine storms and compensatory GDF15 upregulation in both liver and macrophages. Hepatocyte-specific GDF15 overexpression attenuated injury through restored mitochondrial integrity, diminished macrophage infiltration, and reduced systemic inflammation, as evidenced by significantly lower levels of circulating TNF-α and IL-6. Mechanistically, GDF15 preserved mitochondrial homeostasis by inducing SMAD7 expression while suppressing HIF-1α accumulation and PKM2 nuclear translocation. Pharmacological HIF-1α/PKM2 inhibition recapitulated GDF15’s protective effects, restoring mitochondrial function and reducing inflammation even in GDF15-deficient models. Clinical analysis of a sepsis patient cohort (n=119) confirmed a significant elevation of circulating GDF15, with its levels strongly correlating with disease severity scores. Critically, SMAD7 activation attenuated HIF-1α accumulation and rescued mitochondrial failure independently of GDF15 status.

**Conclusion:**

GDF15 orchestrates sepsis tolerance through the SMAD7-HIF-1α axis, preserving mitochondrial integrity while resolving metabolic-inflammatory dysregulation, notably by suppressing the release of pro-inflammatory cytokines such as TNF-α and IL-6. This study identifies GDF15 as a central guardian of mitochondrial-immune homeostasis in sepsis, positioning it as both a robust severity biomarker and a promising therapeutic target for mitochondrial resuscitation.

## Introduction

Sepsis remains a leading cause of mortality in intensive care units, characterized by dysregulated host responses to infection that progress from hyperinflammation to multi-organ failure. The hepatic compartment plays a critical role in this pathology, with mitochondrial dysfunction emerging as a key driver of disease progression. Crucially, we distinguish this dysfunction (defined as bioenergetic impairment and signaling dysregulation) from irreversible mitochondrial or cellular death, positioning it as a target for resuscitation. Despite advances in antimicrobial therapies, mortality rates approach 20% due to limited interventions targeting organ-protective pathways, creating an urgent need for mechanistic insights into endogenous cytoprotective systems (1, 2).

Growth Differentiation Factor 15 (GDF15), a stress-responsive growth differentiation factor, has gained recognition as a pleiotropic regulator of metabolic adaptation during inflammatory challenges. Its expression correlates with disease severity across multiple pathologies including cardiovascular disease, cancer cachexia, and critical illness. GDF15 was initially characterized to signal through the hindbrain-restricted GFRAL receptor, activating neuronal circuits that modulate peripheral metabolic responses (3). Crucially, GDF15 demonstrates organ-protective potential through preservation of mitochondrial function and attenuation of inflammatory cascades, though its mechanistic integration in sepsis remains incompletely characterized (4–6).

The present study establishes GDF15 as a central coordinator of mitochondrial-immune homeostasis during endotoxemia. We demonstrate that LPS-induced hepatic injury triggers compensatory GDF15 upregulation in both hepatocytes and macrophages, with spatiotemporal induction patterns corresponding to cytoprotective responses. Through gain- and loss-of-function approaches, we identify that GDF15 preserves mitochondrial integrity via SMAD7-mediated suppression of HIF-1α, subsequently attenuating PKM2 nuclear translocation. Pharmacological validation confirms that SMAD7 activation replicates GDF15’s hepatoprotective effects, while targeted inhibition of HIF-1α or PKM2 rescues mitochondrial dysfunction in GDF15-deficient models. These findings delineate a novel regulatory axis wherein GDF15 orchestrates metabolic-immune integration, positioning it as both a prognostic biomarker and therapeutic target for sepsis-induced organ injury.

## Methods

### Experimental models and ethical compliance​

All animal procedures were conducted in accordance with the NIH Guide for the Care and Use of Laboratory Animals (8th edition) and approved by the Institutional Animal Care Committee (IACUC Approval No. No.2024-0802D). Male C57BL/6J mice (6–8 weeks old; Shulaibao Biotechnology, Wuhan, China) were housed under specific pathogen-free conditions with controlled temperature (22°C ± 1°C), humidity (55% ± 5%), and 12-hour light/dark cycles. RAW264.7 macrophages (ATCC TIB-71) were routinely verified for mycoplasma contamination using the MycoAlert PLUS kit (Lonza, LT07-710).

### Reagents and molecular tools

Pharmacological agents included: HIF-1α inhibitor BAY 87-2243 [MedChemExpress, CAS 1227158-85-1; a potent and selective inhibitor of mitochondrial complex I that effectively suppresses HIF-1α protein accumulation under normoxic and hypoxic conditions (7)], PKM2 inhibitor Shikonin [MedChemExpress, CAS 54952-43-1; a specific inhibitor that binds to the PKM2 subunit, suppressing its enzymatic activity and nuclear translocation, with well-documented anti-inflammatory effects (8)], and SMAD7 activator Asiaticoside (MedChemExpress, 16830-15-2; a triterpenoid compound known to upregulate SMAD7 expression and ameliorate inflammation in macrophage models (9)).

### Cell culture and interventions​

RAW264.7 cells were cultured in DMEM (Gibco, 11965092) supplemented with 10% FBS (Gibco, 16000-044) and 1% penicillin/streptomycin (Gibco, 15140122) at 37 °C/5% CO_2_. For experiments, cells underwent serum starvation (1% FBS, 12 h) before transduction with rAAV8-mGdf15 (MOI = 10^4^, 12 h) or transfection with si-GDF15 (50 nM, Lipofectamine 3000). LPS stimulation (200 ng/mL, 24 h) was preceded by 2-hour pretreatment with BAY 87-2243 (10 nM) or Shikonin (20 μM).

### Quantitative real-time PCR

Total RNA was extracted from RAW264.7 cells using TRIzol reagent (Invitrogen) according to the manufacturer’s instructions. RNA concentration and purity were assessed spectrophotometrically. First-strand cDNA was synthesized from 1 µg of total RNA using a PrimeScript RT reagent Kit with gDNA Eraser (Takara). Quantitative PCR was performed using TB Green Premix Ex Taq II (Takara) on a QuantStudio 5 Real-Time PCR System (Applied Biosystems). The thermal cycling conditions were: 95°C for 30 sec, followed by 40 cycles of 95°C for 5 sec and 60°C for 34 sec. Gene expression levels were normalized to the endogenous reference gene β-actin, and relative quantification was calculated using the 2^-ΔΔCt method, with the untreated control group set as the calibrator. The primer sequences used were as follows:

Mouse Gfral forward: 5’-TTCCTGGCTGTTACGTTAAGC-3’,
Mouse Gfral reverse: 5’-GCCATTTGCATCAATCAAGCA-3’;
Mouse β-actin forward: 5’-CCTGACAACGTGCGGAAATG-3’,
Mouse β-actin reverse: 5’-CTTCTGCAACTCAACCACTCC-3’.

### Animal experimental design

Adult male C57BL/6 mice were maintained under specific pathogen-free conditions with a 12-h light/dark cycle and provided with food and water ad libitum. The experimental protocol was as follows. Mice first received an intravenous injection of rAAV8-CMV-mGdf15 (1×10¹¹ viral particles) or a control vector. Seven days after viral transduction, endotoxemia was induced by intraperitoneal injection of LPS (10 mg/kg) under anesthesia with 2% isoflurane. Where applicable, pharmacological inhibitors (BAY 87-2243, 5 mg/kg; Shikonin, 10 mg/kg) were administered intraperitoneally 2 hours prior to the LPS challenge. At 24 hours post-LPS administration, mice were euthanized by cervical dislocation. Peripheral blood was collected via cardiac puncture immediately thereafter, followed by tissue collection.

At the experimental endpoint, euthanasia was performed humanely in accordance with the American Veterinary Medical Association (AVMA) Guidelines for the Euthanasia of Animals (2020 Edition). This was achieved by exposure to gradually filled carbon dioxide at a flow rate of 30–70% of the chamber volume per minute. Death was confirmed by the cessation of breathing and cardiac arrest, followed by cervical dislocation as a secondary measure.

### Molecular phenotyping

Western blotting utilized RIPA lysis buffer (Beyotime, P0013B) with protease/phosphatase inhibitors. Proteins (30 μg/lane) were separated on 10% SDS-PAGE gels, transferred to PVDF membranes (Millipore, IPVH00010), and probed with the following primary antibodies: rabbit anti-mouse UQCRC1 (Proteintech, Cat. No. 21705-1-AP, 1:1000), rabbit anti-mouse GDF15 (Proteintech, Cat. No. 27455-1-AP, 1:1000), rabbit anti-mouse HIF-1α (Proteintech, Cat. No. 20960-1-AP, 1:1000), rabbit anti-mouse SMAD7 (Proteintech, Cat. No. 725840-1-AP, 1:1000), mouse anti-mouse PKM2 (Proteintech, Cat. No. 60268-1-Ig, 1:1000), rabbit anti-mouse H3 (Proteintech, Cat. No. 17168-1-AP, 1:5000) and mouse anti-mouse β-actin (Proteintech, Cat. No. 66009-1-Ig, 1:5000). All antibodies were obtained from Proteintech Group (Wuhan, China). Subcellular fractionation was performed using the Minute Cytosolic/Nuclear Kit (beyotime, Cat.No.: P0027). For immunofluorescence, after 4% PFA fixation and 0.5% Triton X-100 permeabilization, cells were incubated with rat anti-mouse F4/80 (Invitrogen, 14-4801-82, 1:200) and mouse anti-mouse PKM2 (Proteintech, Cat. No. 60268-1-Ig, 1:100). Images were quantified via Imaris 9.7 (Bitplane).

### Biochemical and functional assays​

Serum cytokines were measured using mouse-specific ELISA kits: TNF-α (JONLNBIO, JLW10484) and IL-6 (JONLNBIO, JL20268). Serum levels of the growth differentiation factor GDF15 were measured using a specific ELISA kit (R&D, DY957). Lactate levels were quantified via the Lactate Colorimetric Assay Kit II (BioVision, K627-100). Apoptosis was evaluated via TUNEL staining (Roche, 11684795910) with five random fields per slide quantified.

### Human patient cohort

This study included a retrospective analysis of 119 septic patients and 91 age- and sex-matched healthy controls. Sepsis was diagnosed according to the Sepsis-3 criteria. Blood samples were collected within 24 hours of diagnosis. Serum GDF15 levels were quantified using a commercial ELISA kit (R&D Systems, DY957). Clinical and laboratory data were obtained from medical records. The study was approved by the Institutional Review Board of Renmin Hospital of Wuhan University (IRB No. WDRY2022-K157), and written informed consent was obtained.

### Statistical analysis​

All quantitative data are presented as mean ± standard error of the mean (SEM). Normality distribution was confirmed using the Shapiro-Wilk test (significance threshold α=0.05). Data conforming to normality assumptions were analyzed with parametric statistical methods: intergroup differences were assessed by unpaired t-test (two groups) or one-way analysis of variance (ANOVA) with Tukey’s *post-hoc* test (multiple groups), while variable associations were evaluated via Pearson correlation analysis. Sample size determination was based on *a priori* power analysis using G*Power 3.1 (α=0.05, power β=0.9), which established a minimum requirement of n=8 per group to detect intergroup differences with a 30% effect size. All statistical analyses were performed using GraphPad Prism version 10.1.2, with statistical significance defined as *P < 0.05*.

## Results​

### LPS challenge induces time-dependent mitochondrial dysfunction and metabolic stress with compensatory GDF15 upregulation in liver injury

**Figure 1:**
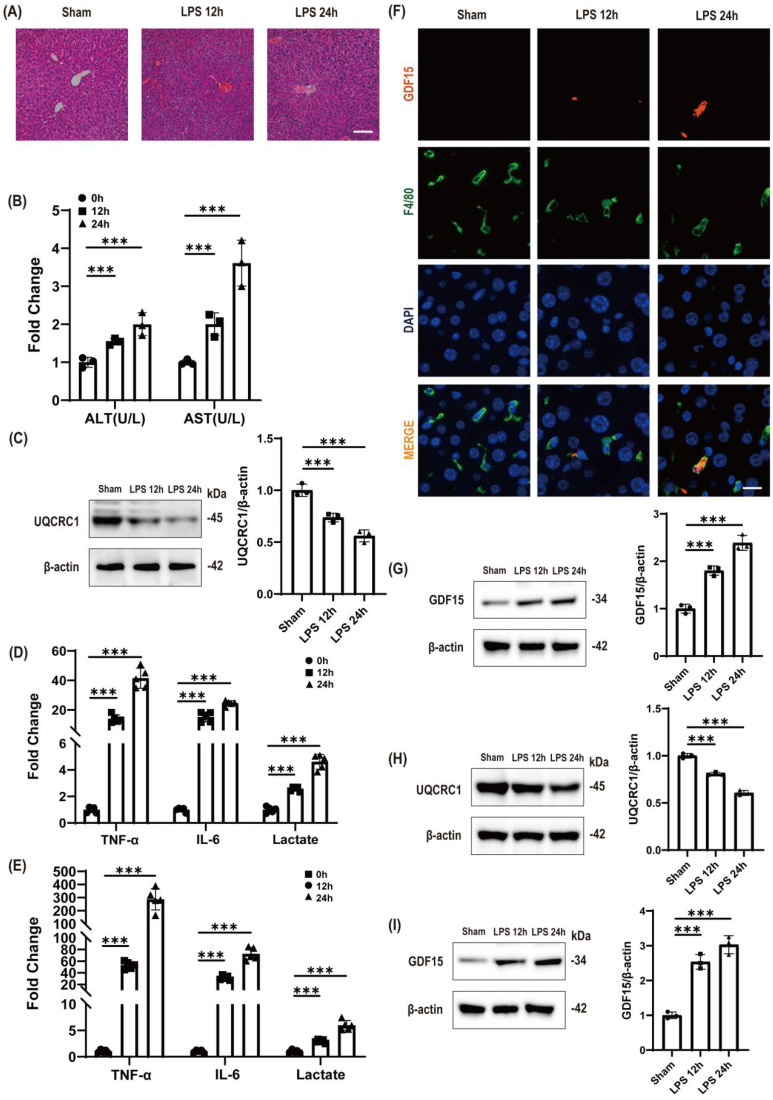
LPS challenge induces time-dependent mitochondrial dysfunction and metabolic stress with compensatory GDF15 upregulation in liver injury **(A)** Temporal liver histopathology (H&E) post-LPS. Scale bar: 50 μm. (Time-resolved hepatic damage progression.) **(B)** Serum ALT/AST kinetics (n = 3). Data: mean ± SD. Study groups and individual replicates are identified in the figure key. ***p < 0.001. (Biomarker-confirmed hepatocyte injury.) **(C)** Progressive loss of hepatic UQCRC1 (mitochondrial complex III core subunit). β-actin: loading control.(Impaired mitochondrial electron transport.) **(D)** Time-elevated serum TNF-α, IL-6, and lactate in mice (n = 5). Study groups and individual replicates are identified in the figure key. ***p < 0.001. (Concomitant systemic inflammation and metabolic stress.) **(E)** Time-elevated cell culture supernatant TNF-α, IL-6, and lactate (n = 5). ***p < 0.001. (Concomitant inflammation and metabolic stress in cell culture system.) **(F)** Spatiotemporal GDF15-F4/80 co-localization in liver. GDF15 (orange), macrophages (F4/80, green), nuclei (DAPI, blue). Scale bar: 20 μm. Representative image showing focal GDF15 induction. Note that the signal is localized to discrete macrophage-enriched inflammatory niches, consistent with the physiological distribution of immune cells in hepatic tissue. **(G)** Hepatic GDF15 accumulation after LPS challenge. β-actin served as a loading control. Note: the detected band at ~34 kDa corresponds to the glycosylated pro-form of GDF15. (Liver-wide stress adaptation response.) **(H)** Persistent UQCRC1 suppression in hepatocytes (validating *in vivo* impairment in C).(Cell-level mitochondrial dysfunction.) **(I)** GDF15 induction in RAW264.7 cells. β-actin: loading control. (Cell-autonomous GDF15 upregulation.).

LPS challenge in endotoxemic mice provoked severe hepatic injury, evidenced by progressive histopathological damage (**Figure 1A**), substantially elevated serum transaminase levels (**Figure 1B**), and significant mitochondrial dysfunction in liver tissue at 24 hours (**Figure 1C**), and systemic inflammatory activation—evidenced by dramatic increases in pro-inflammatory cytokines (TNF-α, IL-6) and metabolic markers at 24 hours (**Figure 1D**). Parallel studies in RAW264.7 macrophages demonstrated LPS-induced mitochondrial impairment (**Figure 1H**) and amplified inflammatory cascades, characterized by robust cytokine secretion and metabolic shifts (**Figure 1E**). Notably, a time-dependent upregulation of GDF15 was consistently observed across both experimental systems, showing progressive induction in liver tissue over 12–24 hours (**Figure 1G**) and significant elevation in macrophages at 24 hours (**Figure 1I**). The observed GDF15 band at approximately 34 kDa corresponds to the glycosylated pro-form of the protein, which is commonly detected in cell and tissue lysates and is consistent with the specifications of the antibody used as well as numerous independent reports (10–12). Spatial co-localization of GDF15-expressing cells within macrophage-dominated inflammatory foci was confirmed by immunofluorescence (**Figure 1F**), delineating an evolutionarily conserved cytoprotective mechanism against LPS-induced tissue damage.

To investigate the receptor basis for GDF15 action in macrophages, we directly examined the expression of its canonical receptor, GFRAL, in RAW264.7 cells using qRT-PCR. We found that GFRAL is constitutively expressed in these cells. Furthermore, its expression level remained stable and was not significantly altered by treatment with LPS, rAAV8-mGdf15, or si-GDF15 (**Supplementary Figure S1**). This confirms the presence of the GDF15 receptor in our cellular model, fulfilling a prerequisite for direct GDF15 signaling.

### Hepatoprotective effects of GDF15 overexpression against LPS-induced injury via mitochondrial function restoration

**Figure 2:**
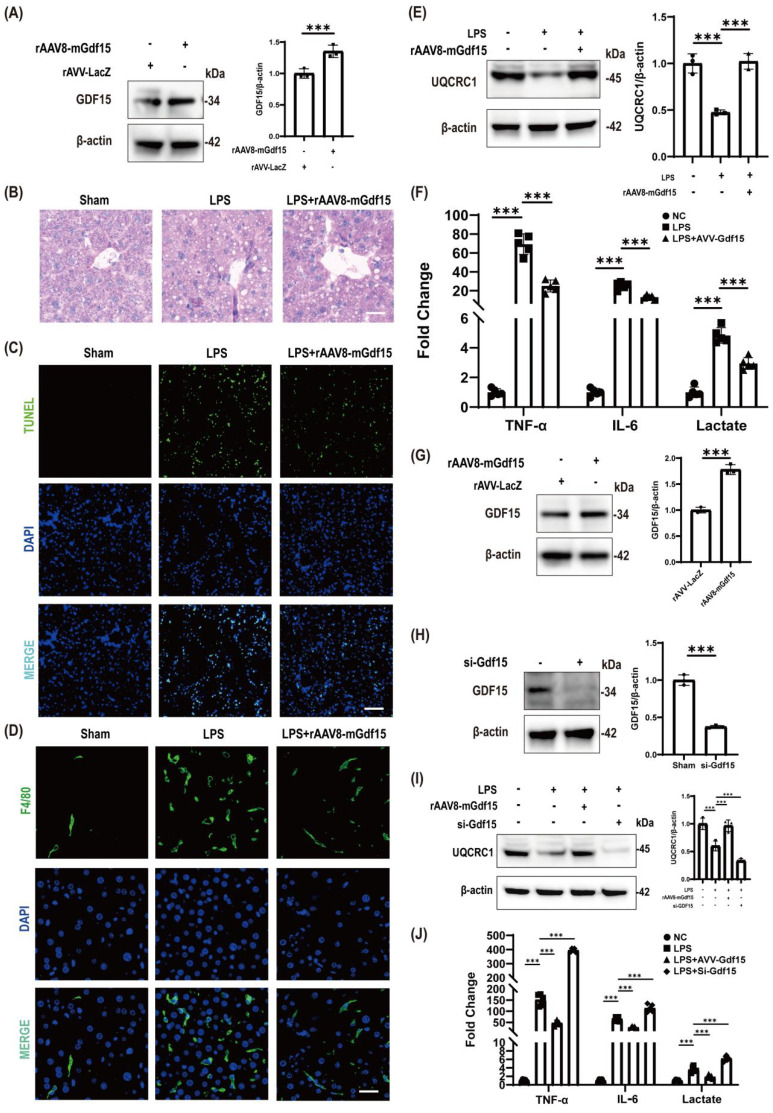
Hepatoprotective effects of GDF15 overexpression against LPS-induced injury via mitochondrial function restoration. **(A)** Robust GDF15 expression in mouse liver following rAAV8-mGdf15 delivery. β-actin: loading control.(rAAV8-mediated hepatic GDF15 overexpression. β-actin: loading control.) **(B)** H&E-stained liver sections: Untreated (NC), LPS-challenged (LPS), and LPS + rAAV8-mGdf15 (LPS+GDF15). Scale bar: 50 μm. (Histopathological rescue by GDF15.) **(C)** TUNEL assay (green) showing apoptotic cell death. DAPI (blue): nuclei. GDF15 significantly reduces apoptosis. Scale bars: 20 μm.(GDF15-mediated suppression of apoptosis. TUNEL cells (green), nuclei (DAPI, blue).) **(D)** Macrophage infiltration (F4/80+, green) attenuated by GDF15 overexpression. DAPI (blue): nuclei. Scale bars: 100 μm.(Inhibition of macrophage recruitment. F4/80+ cells (green), nuclei (DAPI, blue).) **(E)** Hepatic UQCRC1 recovery with GDF15. β-actin: loading control.(Mitochondrial complex III rescue.) **(F)** Serum TNF-α, IL-6, and lactate levels (n = 5). Study groups and individual replicates are identified in the figure key. ***p < 0.001. (Systemic inflammation and metabolic stress reversal.) **(G)** rAAV8-mGdf15 elevates GDF15 in RAW264.7. β-actin: loading control.(Macrophage-targeted GDF15 overexpression.) **(H)** Effective GDF15 knockdown (si-GDF15) in RAW264.7. β-actin: loading control.(GDF15 knockdown efficiency.) **(I)** UQCRC1 expression in RAW264.7: Loss of GDF15 (si-GDF15) exacerbates LPS-induced UQCRC1 suppression, while GDF15 restores it.(GDF15-dependent mitochondrial protection in macrophages.) **(J)** Inflammatory (TNF-α, IL-6) and metabolic (lactate) markers in RAW264.7 supernatant (n = 5 independent experiments). GDF15 inhibits LPS-induced release; si-GDF15 amplifies it. Study groups and individual replicates are identified in the figure key.(GDF15-modulated macrophage inflammatory output.).

rAAV8-mediated GDF15 overexpression in mouse liver (**Figure 2A**) conferred significant protection against LPS-induced injury, as demonstrated by attenuated histopathological damage (**Figure 2B**) and suppressed apoptosis (**Figure 2C**). This hepatoprotection coincided with reduced macrophage infiltration (**Figure 2D**) and restored mitochondrial electron transport function (**Figure 2E**). Systemically, GDF15 overexpression significantly attenuated LPS-triggered elevations in serum inflammatory mediators, most notably TNF-α and IL-6 (**Figure 2F**).

Parallel macrophage-specific studies revealed that rAAV8-driven GDF15 overexpression (**Figure 2G**) attenuated LPS-induced mitochondrial impairment (**Figure 2I**), while effective GDF15 knockdown (**Figure 2H**) exacerbated mitochondrial dysfunction (**Figure 2I**). Consequently, GDF15-overexpressing macrophages exhibited suppressed inflammatory mediator release, contrasting with amplified responses upon GDF15 depletion (**Figure 2J**), collectively establishing GDF15-dependent regulation of macrophage inflammatory output through mitochondrial protection.

### GDF15 preserves mitochondrial homeostasis in LPS-stimulated macrophages through dual regulation of SMAD7 and PKM2 pathways

**Figure 3:**
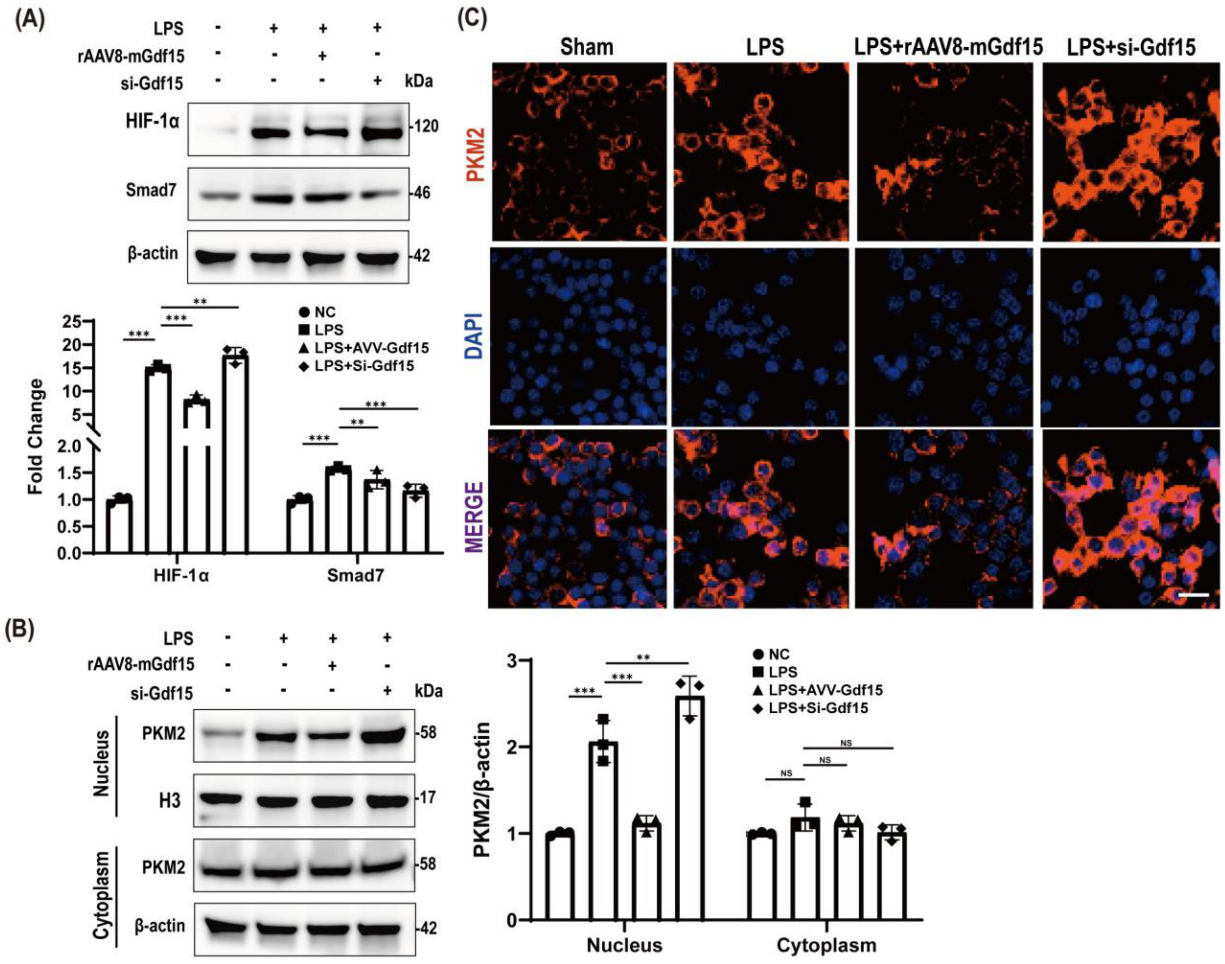
GDF15 preserves mitochondrial homeostasis in LPS-stimulated macrophages through dual regulation of SMAD7 and PKM2 pathways. **(A)** HIF-1α and SMAD7 expression in RAW264.7 macrophages across conditions: Untreated, LPS, LPS with rAAV8-mGdf15 overexpression (LPS+GDF15), and LPS with GDF15 knockdown (si-GDF15). β-actin: loading control.(HIF-1α suppression and SMAD7 induction by GDF15.) **(B)** Cytosolic and nuclear PKM2 protein levels. Lamin B1 (nuclear) and α-tubulin (cytosolic) markers validate fractionation efficiency. Study groups and individual replicates are identified in the figure key.(PKM2 subcellular redistribution modulated by GDF15.) **(C)** Immunofluorescence of PKM2 (red) and nuclei (DAPI, blue). Arrows indicate nuclear PKM2 accumulation. Scale bar: 15 μm.(Nuclear PKM2 enrichment upon LPS challenge mitigated by GDF15 and exacerbated by GDF15 knockdown.).

In RAW264.7 macrophages, GDF15 overexpression significantly elevated SMAD7 expression while suppressing HIF-1α accumulation—effects significantly attenuated by GDF15 knockdown (**Figure 3A**). Concurrently, GDF15 attenuated LPS-induced nuclear translocation of PKM2, reducing nuclear accumulation (**Figure 3B**). Immunofluorescence confirmed these spatial dynamics: GDF15 overexpression mitigated LPS-driven nuclear PKM2 enrichment, whereas GDF15 knockdown exacerbated it (**Figure 3C**). Collectively, these results demonstrate that GDF15 modulates HIF-1α and SMAD7 expression while regulating PKM2 nuclear accumulation in macrophages.

### HIF-1α and PKM2 are critical effectors of GDF15-driven mitochondrial protection and anti-inflammatory responses

**Figure 4:**
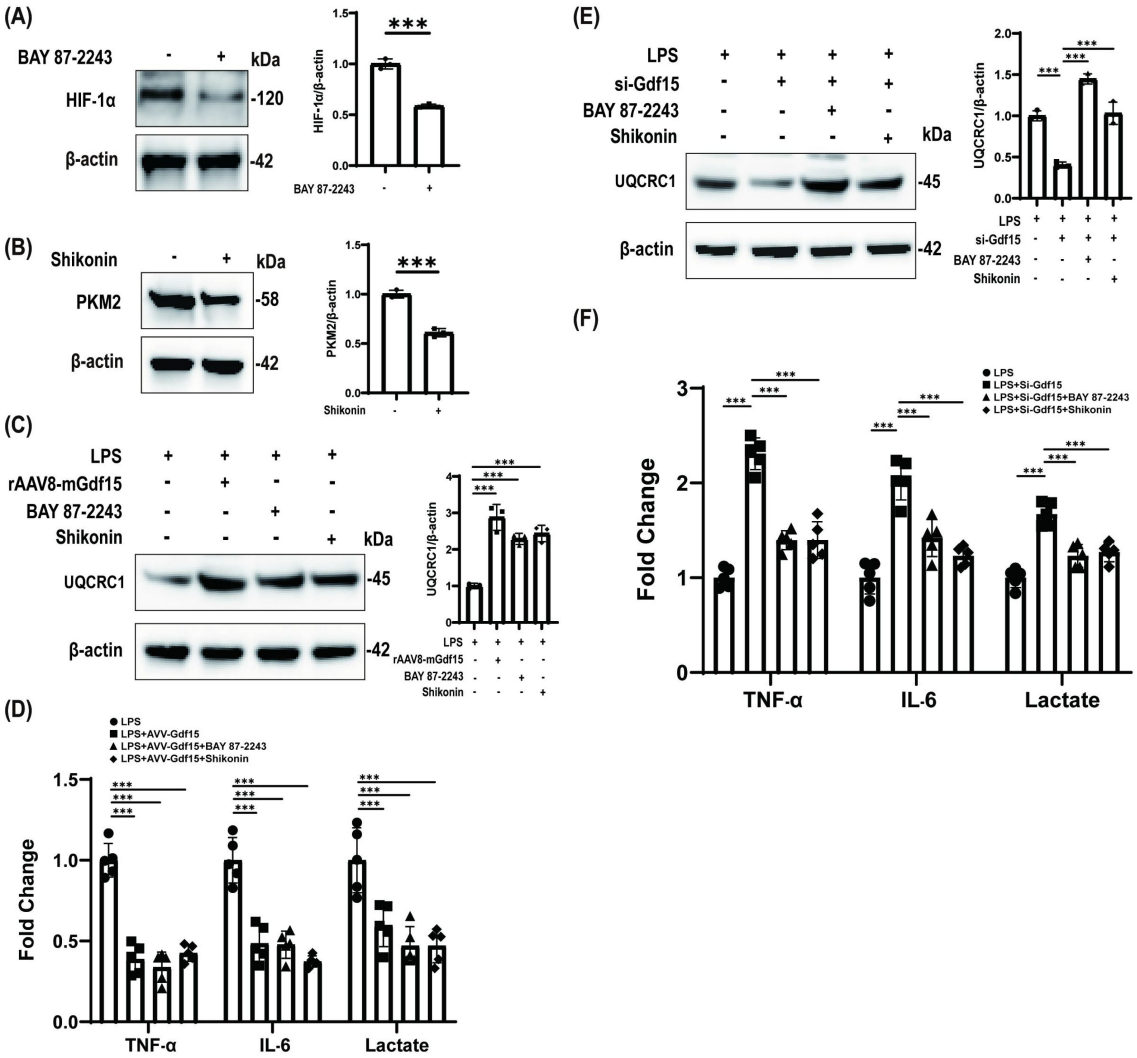
HIF-1α and PKM2 are critical effectors of GDF15-driven mitochondrial protection and anti-inflammatory responses. **(A)** HIF-1α inhibition by BAY 87-2243 (5 μM, 24 h). β-actin: loading control.(Pharmacological HIF-1α blockade.) **(B)** PKM2 inhibition by Shikonin (2 μM, 24 h). β-actin: loading control.(PKM2 activity suppression.) **(C)** UQCRC1 recovery in LPS-injured macrophages treated with: GDF15 overexpression, HIF-1α inhibitor (BAY), or PKM2 inhibitor (Shikonin). β-actin: loading control.(Mitochondrial complex III rescue via HIF-1α/PKM2 inhibition mirrors GDF15 effects.) **(D)** Inflammatory (TNF-α, IL-6) and metabolic (lactate) markers in cell supernatant (n = 5). Study groups and individual replicates are identified in the figure key. ***p < 0.001.(HIF-1α/PKM2 targeting replicates GDF15-mediated anti-inflammatory and metabolic homeostasis.) **(E)** UQCRC1 expression under GDF15 loss-of-function: si-GDF15 alone vs. combined with BAY 87–2243 or Shikonin. β-actin: loading control. Study groups and individual replicates are identified in the figure key.(Mitochondrial rescue in GDF15-deficient macrophages requires HIF-1α/PKM2 inhibition.) **(F)** Supernatant cytokines and lactate in si-GDF15 macrophages with/without inhibitors (n = 5). ***p < 0.001. (Inflammation reversal in GDF15-knockdown macrophages depends on HIF-1α/PKM2 blockade.).

Pharmacological targeting of HIF-1α and PKM2 recapitulated GDF15-mediated mitochondrial protection and anti-inflammatory effects in LPS-stimulated macrophages. HIF-1α inhibition significantly suppressed HIF-1α accumulation (**Figure 4A**), while PKM2 inhibition attenuated PKM2 activity (**Figure 4B**). Crucially, both interventions mirrored GDF15 overexpression in restoring mitochondrial complex III integrity (**Figure 4C**) and suppressing inflammatory cytokine release and metabolic dysregulation (**Figure 4D**).

In GDF15-deficient macrophages, mitochondrial impairment was rescued by HIF-1α or PKM2 inhibition (**Figure 4E**), with concordant reversal of hyperinflammation (**Figure 4F**). Collectively, these data establish GDF15 as a master regulator of endotoxemic homeostasis, orchestrating mitochondrial stabilization and inflammation resolution across hepatic and immune compartments to disrupt sepsis-associated metabolic-inflammatory injury.

### SMAD7 activation suppresses HIF-1α to mediate GDF15-dependent mitochondrial protection in LPS-challenged macrophages

**Figure 5:**
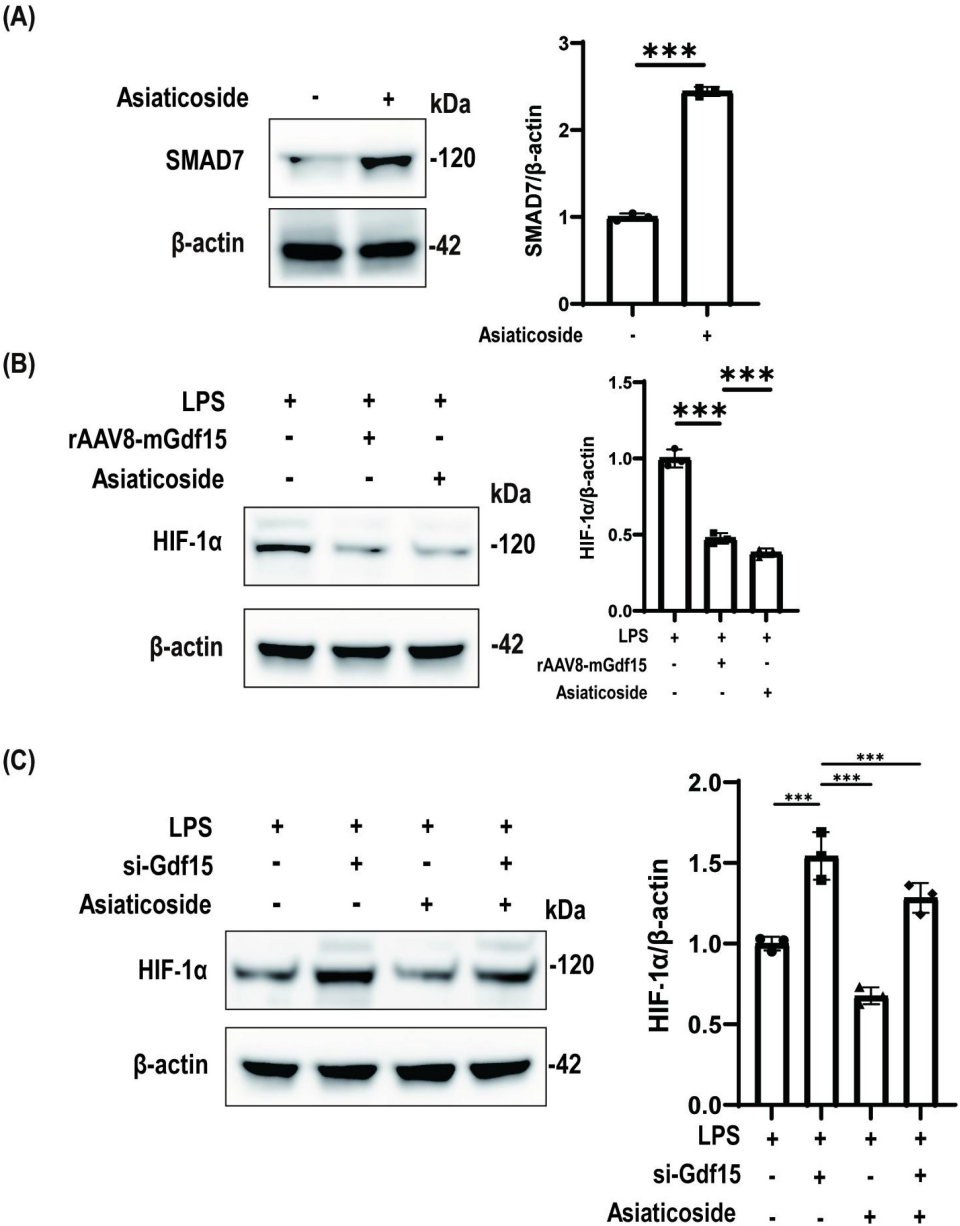
SMAD7 activation suppresses HIF-1α to mediate GDF15-dependent mitochondrial protection in LPS-challenged macrophages. **(A)** Pharmacological SMAD7 activation by Asiaticoside (20 μM, 48 h). β-actin: loading control. **(B)** HIF-1α expression under LPS challenge: LPS alone, LPS + AVV-GDF15, or LPS + SMAD7 activation (Asiaticoside). β-actin: loading control. **(C)** HIF-1α modulation across conditions: LPS, LPS + si-GDF15, LPS + Asiaticoside, or LPS + si-GDF15 + Asiaticoside. β-actin: loading control.

Pharmacological activation of SMAD7 by Asiaticoside significantly induced SMAD7 expression (**Figure 5A**). In LPS-stimulated macrophages, both GDF15 overexpression and SMAD7 activation comparably attenuated HIF-1α accumulation (**Figure 5B**). GDF15 deficiency exacerbated HIF-1α upregulation, which was significantly rescued by SMAD7 activation (**Figure 5C**). Collectively, these results establish SMAD7 as the non-redundant executor of HIF-1α suppression within the GDF15 cytoprotective axis.

### Clinical validation of GDF15 as a sepsis severity biomarker

To translate our mechanistic findings to the clinical setting, we analyzed a cohort of 119 septic patients and 91 healthy controls (see Methods). Clinical analysis of 119 septic patients and 91 healthy controls confirmed profound dysregulation of inflammatory and metabolic parameters in sepsis (**Table 1**). Septic patients exhibited significantly elevated levels of white blood cells (WBC), C-reactive protein (CRP), serum amyloid A (SAA), procalcitonin (PCT), lactate, and glucose, alongside thrombocytopenia and hepatic injury. Most notably, GDF15 levels were markedly higher in septic patients, affirming its clinical relevance as a sepsis-associated biomarker.

**Table 1:** Characteristics of septic patients and healthy controls.

Characteristics	Septic patients (n = 119)	Healthy controls (n = 91)	χ²/t/Z	p value
Age(years)	67.37(65.11,69.63)	66.41(63.73,69.08)	0.548	0.585
WBC(×10^9^/L)	13.95(12.54,15.35)	11.64(10.44,12.83)	-1.966	0.049
RBC(×10¹²/L)	3.4(3.23,3.57)	3.37(3.16,3.58)	-0.689	0.491
CRP(mg/L)	106.18(91.3,121.06)	58.13(44.93,71.33)	-4.786	<0.001
SAA(mg/L)	225.16(206,244.32)	156(129.83,182.18)	-3.918	<0.001
PCT(ng/ml)	24.56(18.41,30.71)	1.28(0.89,1.67)	-8.395	<0.001
Glu(mmol/L)	10.49(9.39,11.58)	8.61(7.71,9.5)	-2.175	0.03
Lactate (mmol/L)	3.08(2.5,3.67)	2.15(1.65,2.66)	-2.699	0.007
PLT(×10^9^/L)	142.92(126.97,158.88)	184.48(161.53,207.44)	-2.679	0.007
SOFA	8.26(7.53,8.99)	4.95(4.28,5.61)	-6.023	<0.001
ALT(U/L)	29.44(24.91,33.96)	19.29(17.31,21.26)	-2.249	0.025
AST(U/L)	51.93(42.82,61.04)	28.18(24.98,31.37)	-3.577	<0.001
TBIL(μmol/L)	23.93(19.27,28.58)	12.33(11.43,13.23)	-3.282	0.001
IL-6(pg/mL)	232.65(186.74,278.55)	6.17(5.38,6.95)	-12.17	<0.001
TNF-α(pg/mL)	252.31(201.06,303.56)	6.19(5.36,7.02)	-11.75	<0.001
GDF15(ng/mL)	4.72(4.09,5.36)	1.69(1.44,1.94)	-7.597	<0.001

**Figure 6:**
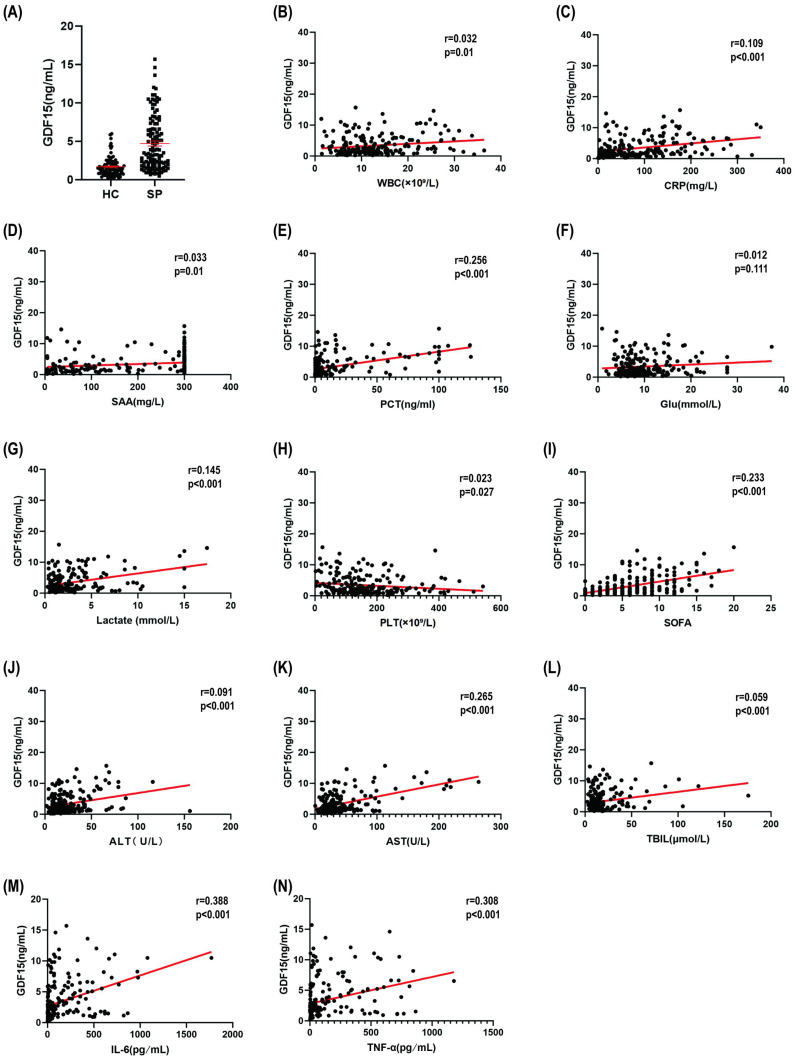
GDF15 correlates with clinical severity in sepsis. **(A)** Circulating GDF15 levels: Healthy controls (HC, n=91) vs. Sepsis patients (SP, n=119). **(B–N)** Correlation network of GDF15 with clinical parameters: WBC, CRP, SOFA, SAA, PCT, Glu, Lactate, PLT, SOFA, ALT, AST, TBIL, IL-6 and TNF-α (n=210). Solid lines represent linear regression fits for visual trend estimation, while r and P values are derived from Spearman’s rank correlation analysis.

Correlation analyses further demonstrated that GDF15 levels were positively associated with multiple markers of systemic inflammation (CRP, PCT, SAA) and organ dysfunction indices (**Figures 6A–L**), including WBC, CRP, and SOFA score. Particularly compelling were its strong correlation with lactate and inverse correlation with platelet count, findings consistent with experimental data implicating GDF15 in metabolic stress adaptation and suppression of megakaryopoiesis. These clinical observations reinforce the role of GDF15 as a critical mediator of inflammatory-metabolic crosstalk in sepsis.

## Discussion

Sepsis is characterized by a self-amplifying cycle where initial hyperinflammation (cytokine storm) induces mitochondrial dysfunction, which in turn exacerbates inflammation, ultimately leading to multi-organ failure. Our study identifies GDF15 as a critical factor that disrupts this pathogenic cycle in the liver. Hepatic injury manifests as vascular rupture, sinusoidal distortion, and neutrophil infiltration (2, 6, 13, 14).

Mitochondrial dysfunction and inflammation form a self-amplifying loop in sepsis pathogenesis. Damaged mitochondria release mtDNA/ROS as DAMPs, activating cGAS-STING-driven interferon responses and NLRP3 inflammasome-mediated IL-1β maturation (15). Conversely, inflammatory cytokines inhibit ETC complexes and induce mPTP opening (16), while immune cell ROS/RNS oxidize mitochondrial components (17). Critically, chronic inflammation suppresses mitophagy, allowing damaged mitochondria to accumulate and escalate mtDAMP signaling. This bidirectional cycle sustains cytokine storms and multi-organ failure (18).

Consistent with prior research, serological profiling identifies GDF15 as a pivotal integrator of metabolic-inflammatory stress in sepsis, showing marked elevation during disease progression (6, 14). Its strong correlations with organ dysfunction (e.g., SOFA score), metabolic acidosis (lactate), and systemic inflammation (PCT/CRP/SAA) establish its utility as a composite biomarker of disease severity. Notably, the absence of correlation with glucose underscores specificity to pathological stress pathways rather than generalized metabolic dysregulation. Clinically, the sepsis serum profile—characterized by elevated WBC, CRP, SAA, PCT, lactate, thrombocytopenia, and hepatic injury—recapitulates LPS-induced mitochondrial dysfunction and cytokine storm phenotypes. The inverse correlation between GDF15 and platelet count corresponds to GDF15-mediated suppression of megakaryopoiesis—a mechanism conserved in macrophage models and indicative of stress-adapted hematopoiesis.

It is important to address the apparent paradox between clinical observations—where elevated GDF15 positively correlates with pro-inflammatory cytokines (**Table 1**, **Figure 6**) (19)—and our mechanistic findings demonstrating GDF15’s anti-inflammatory function. This co-elevation likely reflects a compensatory stress response rather than a causative role in driving inflammation. As a stress-inducible cytokine (often termed a ‘mitokine’), GDF15 expression is triggered by the very inflammatory and metabolic insults (e.g., LPS, mitochondrial stress) that it serves to resolve. Therefore, high circulating GDF15 in septic patients represents a physiological negative feedback loop or ‘disease tolerance’ mechanism attempting to dampen the cytokine storm, analogous to the rise of anti-inflammatory mediators during severe infection. While the correlation reflects disease severity (the magnitude of the insult), our gain-of-function data (**Figure 2**) isolate the causation, confirming that GDF15 actively suppresses the inflammatory cascade.

The reviewer raises a pertinent question regarding the comparative utility of GDF15 against other circulating markers of mitochondrial (dys)function, such as fibroblast growth factor 21 (FGF21) and acylcarnitines. GDF15, FGF21, and acylcarnitines are all established as biomarkers of mitochondrial stress, albeit with distinct biological origins, specificities, and clinical implications. FGF21 is a hepatokine primarily induced by fasting, metabolic stress, and genetic oxidative phosphorylation defects, and is considered a relatively specific biomarker for primary mitochondrial diseases (20). In contrast, GDF15 is a more pleiotropic factor, described as a “mitochondrial stress hormone”, that is broadly upregulated in response to diverse cellular stresses, including mitochondrial dysfunction, integrated stress response activation, and inflammatory states (21, 22). This broader inducibility may, on one hand, reduce its specificity for primary mitochondrial disorders compared to FGF21, but on the other hand, enhances its value as a composite biomarker of systemic stress and disease severity in complex, multi-factorial conditions like sepsis (23). In fact, a recent hospital-wide study demonstrated that GDF15 exhibits good clinical utility in predicting mitochondrial DNA-related disorders with a sensitivity of 76% and specificity of 88%, while its elevation in non-mitochondrial disorders was low (11-13%) (23). Notably, co-elevation of GDF15 and FGF21 is considered a characteristic signature of mitochondrial dysfunction, suggesting their complementary rather than mutually exclusive roles (24). Compared to the technically complex acylcarnitine profiling by mass spectrometry—which provides detailed insights into specific metabolic blocks within fatty acid oxidation—GDF15 offers practical advantages as a single, stable protein that can be robustly quantified using standardized, widely available immunoassays (e.g., ELISA). Therefore, while FGF21 may offer higher specificity for canonical mitochondrial diseases, and acylcarnitines provide mechanistic metabolic detail, GDF15 emerges as a superior pragmatic and prognostic biomarker in the context of sepsis. It effectively integrates the overall burden of mitochondrial and inflammatory stress, correlates strongly with organ dysfunction and mortality, and is readily translatable to routine clinical laboratory settings.

LPS orchestrates GDF15 production in hepatic immune cells through a coordinated multi-pathway mechanism involving both neuroendocrine signaling and cellular metabolic reprogramming. LPS stimulation activates hepatic β_3_-adrenergic receptors (3), triggering cAMP-PKA signaling that drives adipose triglyceride lipase (ATGL)-mediated lipolysis (3, 25), with palmitate serving as a key free fatty acid mediator; this metabolite activates PPARγ in M2-like macrophages to directly stimulate Gdf15 transcription (25). Concurrently, LPS independently activates the Keap1-NRF2 axis in microglia (5), bypassing classical TLR4 downstream effectors to drive Gdf15 promoter activity (5). These processes are further amplified by LPS-induced metabolic reprogramming (26), which shifts macrophages toward oxidative phosphorylation (OXPHOS) dominance through enhanced mitochondrial complex I/III/V activity (26), thereby providing the energetic substrate required for sustained GDF15 synthesis. Crucially, this mechanism exhibits tissue-specific regulation, with β-AR-PPARγ dominating peripheral responses while NRF2 governs central modulation (3, 5, 25). The resultant GDF15 establishes a self-amplifying loop by suppressing NF-κB-mediated pro-inflammatory cytokine release, which otherwise inhibits its production (26, 27), ultimately coordinating hepatic immunometabolic adaptation during septic stress.

In sepsis patients, serum GDF15 levels are significantly elevatedand exhibit a strong inverse correlation with mortality risk (non-survivors vs. survivors: P<0.001), positioning it as a prognostic biomarker for disease severity (3). Mechanistic studies reveal that inflammatory stimuli (e.g., LPS) induce robust hepatic and renal GDF15 expression, activating GFRAL receptors in the hindbrain’s area postrema to trigger hepatic sympathetic outflow. This neural signaling drives hepatic triglyceride secretion via β-adrenergic pathways, a metabolic adaptation critical for maintaining cardiac energetics during inflammatory stress (28). Experimental models demonstrate that GDF15 neutralization disrupts this axis, causing cardiac dysfunction, thermoregulatory failure, and 80% increased mortality. Crucially, therapeutic interventions with recombinant GDF15 reverse metabolic collapse, improving survival by >50% even when administered 6 hours post-infection, while exogenous triglyceride supplementation (Intralipid) rescues cardiac damage, collectively validating GDF15’s role in sustaining the brain-liver-heart metabolic axis essential for sepsis tolerance (3).

The canonical receptor for GDF15, GFRAL, is well-established for its role in the hindbrain to mediate central metabolic effects (28). In the present study, we provide direct experimental evidence that GFRAL is also constitutively expressed in murine RAW264.7 macrophages, and its expression is not regulated by inflammatory or GDF15-modulating stimuli (**Supplementary Figure S1**). This finding identifies a potential direct signaling pathway for GDF15 within immune cells in our sepsis model. However, a key limitation of this study is that we did not perform functional loss-of-experiments for GFRAL to definitively prove that the observed cytoprotective effects are strictly dependent on this receptor. Future studies employing genetic ablation of GFRAL in macrophages are warranted to establish its functional necessity. It is noteworthy that GDF15 can also signal through context-dependent, GFRAL-independent mechanisms, as demonstrated in other physiological contexts (29, 30). Therefore, while the presence of GFRAL offers a plausible mechanism, the relative contribution of GFRAL-dependent versus alternative pathways in mediating the protective effects of GDF15 during sepsis remains an important area for future investigation.

In conjunction with the robust elevation of classic pro-inflammatory cytokines (IL-6 and TNF-α) in our septic patient cohort (**Table 1**), the strong correlations observed between GDF15 and other established clinical markers of systemic inflammation—including C-reactive protein (CRP), procalcitonin (PCT), and serum amyloid A (SAA)—provide robust evidence linking GDF15 to the overall inflammatory burden in sepsis. The parallel dysregulation of GDF15, IL-6, and TNF-α underscores the intertwined nature of metabolic stress and cytokine storm in sepsis pathogenesis. CRP and PCT, in particular, are widely used in clinical practice to diagnose and monitor the severity of septic inflammation. Therefore, the significant associations reported here effectively quantify the relationship between GDF15 and the patient’s inflammatory status. Future studies incorporating a broader panel of cytokines will be valuable to delineate more specific immune cell-activation profiles associated with GDF15 elevation.

This study establishes GDF15 as a central orchestrator of mitochondrial-immune homeostasis in sepsis, coordinating cytoprotection through the novel SMAD7-HIF-1α-PKM2 signaling axis. Based on the integrated evidence, GDF15 orchestrates HIF-1α generation primarily through a GFRAL-Smad7-TGF-β/PI3K axis, operating independently of hypoxia (31, 32). GDF15 binding to its receptor GFRAL induces Smad7 expression in fibroblasts (31), which subsequently suppresses TGF-β signaling by competitively inhibiting Smad2/3 phosphorylation and nuclear translocation (33). This Smad7-mediated attenuation of TGF-β signaling intercepts TGF-β’s capacity to activate PI3K-dependent pathways that drive HIF-1α protein translation under normoxic conditions (34). Consequently, the GDF15-initiated Smad7 induction creates a regulatory circuit that diminishes HIF-1α synthesis by disrupting TGF-β/PI3K signaling, without altering HIF-1α mRNA stability or degradation kinetics (34, 35). This mechanism highlights a GDF15-regulated pathway for HIF-1α modulation during fibrogenesis, with notable isoform specificity as evidenced by preferential HIF-2α (rather than HIF-1α) target gene activation in pathological microenvironments (27, 34).

Spatiotemporal induction of GDF15—with peak expression in hepatocytes and macrophages post-LPS and specific localization within macrophage-enriched inflammatory foci (**Figure 1E**)—reveals its role as an endogenous stress sentinel. This response directly counteracts LPS-induced hepatic mitochondrial dysfunction, metabolic crisis, and systemic cytokine storms. Mechanistically, GDF15 initiates a hierarchical cascade: SMAD7 upregulation drives HIF-1α suppression, which subsequently attenuates PKM2 nuclear translocation, ultimately restoring mitochondrial integrity and resolving inflammation.

Crucially, GDF15^+^/F4/80^+^ co-localization in inflammatory foci demonstrates microenvironment-specific defense, where macrophage-derived GDF15 preferentially buffers localized hyperinflammation, a phenomenon consistent with our *in vitro* finding that macrophage GDF15 potently suppresses LPS-induced TNF-α and IL-6 secretion (**Figures 2M, N**). Hepatocyte GDF15 preserves structural integrity and restores mitochondrial complex III function. In macrophages, GDF15 overexpression reverses LPS-induced mitochondrial impairment while suppressing cytokine release. It should be noted that this study focused on elucidating the early cytoprotective mechanism and did not evaluate the impact of GDF15 modulation on long-term animal survival, which constitutes an important avenue for future investigation.

In addressing the specificity of the pharmacological tools used, we acknowledge that inhibitors such as BAY 87-2243 (HIF-1α) and Shikonin (PKM2) may have off-target effects. However, the convergence of evidence from both pharmacological and genetic approaches significantly strengthens the specificity of our conclusions. The consistent phenotypic rescue observed—whereby inhibition of HIF-1α or PKM2 counteracted the mitochondrial dysfunction and hyper-inflammation in GDF15-deficient cells—provides compelling evidence that the effects we attribute to this axis are not likely mere artifacts of inhibitor off-target effects. Specifically, SMAD7 activation by Asiaticoside replicated GDF15-mediated HIF-1α suppression and rescued GDF15-deficient phenotypes, while HIF-1α and PKM2 inhibitors recapitulated the mitochondrial protection and anti-inflammatory effects of GDF15.

Differential induction magnitudes between hepatocytes and macrophages define tissue-specific vulnerability thresholds. The presymptomatic GDF15 surge preceding lactate peaks enables prognostic stratification and microenvironment-targeted interventions. Compared to conventional sepsis targets, the GDF15-SMAD7 axis provides superior multimodal protection by synchronizing mitochondrial preservation and inflammation resolution within an early intervention window while avoiding systemic immunosuppression. However, the translation of GDF15 as a therapeutic target warrants caution due to its pleiotropic nature. While our data demonstrate acute hepatoprotection, chronic GDF15 elevation is well-documented to drive cachexia and anorexia. Furthermore, as our study focused on acute mechanistic resolution (24 hours) without assessing long-term survival rates, these therapeutic implications should be viewed as hypothesis-generating. Future interventions must carefully balance the acute mitochondrial benefits against potential chronic deleterious effects.

In conclusion, GDF15 orchestrates a conserved cytoprotective program against septic injury through the SMAD7-HIF-1α-PKM2 axis, simultaneously dismantling the pathological triad of mitochondrial failure, bioenergetic crisis, and cytokine storm. This positions GDF15 as both a predictive biomarker and highlighting its potential relevance in strategies targeting mitochondrial resuscitation.

## Data Availability

The raw data supporting the conclusions of this article will be made available by the corresponding authors, without undue reservation.
