# Supplementary material for: GDF15 orchestrates mitochondrial-immune crosstalk via SMAD7-HIF-1α-PKM2 cascade to attenuate septic liver injury
Source: Front Immunol. 2026 Jan 22;16:1712741. doi: 10.3389/fimmu.2025.1712741 (PMC12872506; doi:10.3389/fimmu.2025.1712741)
Supplement: Supplementary file 1 [file Image1.pdf]

(A)

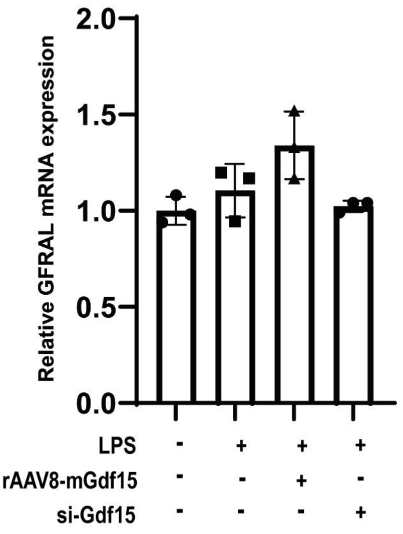

Supplementary Figure S1

Constitutive expression of GFRAL in RAW264.7 macrophages.

Relative mRNA levels of GFRAL were determined by qRT-PCR in cells under the indicated conditions: NC, LPS stimulation, rAAV8-mGdf15, or si-GDF15). Data were normalized to β-actin and are presented as the mean fold change relative to the NC group (dashed line at 1.0) ± SEM (n=3 independent experiments). No significant differences were observed among the groups (one-way ANOVA, ns, not significant).
