# Supplementary figures and images for: GDF15 orchestrates mitochondrial-immune crosstalk via SMAD7-HIF-1α-PKM2 cascade to attenuate septic liver injury
Source: Front Immunol. 2026 Jan 22;16:1712741. doi: 10.3389/fimmu.2025.1712741 (PMC12872506; doi:10.3389/fimmu.2025.1712741)

F1-C

F2-E

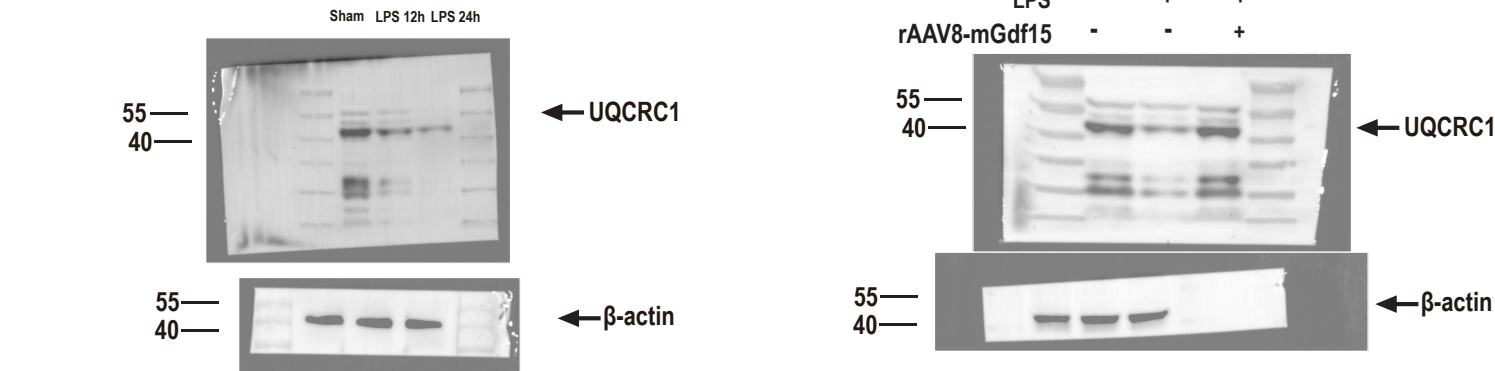

F1-G

F2-G

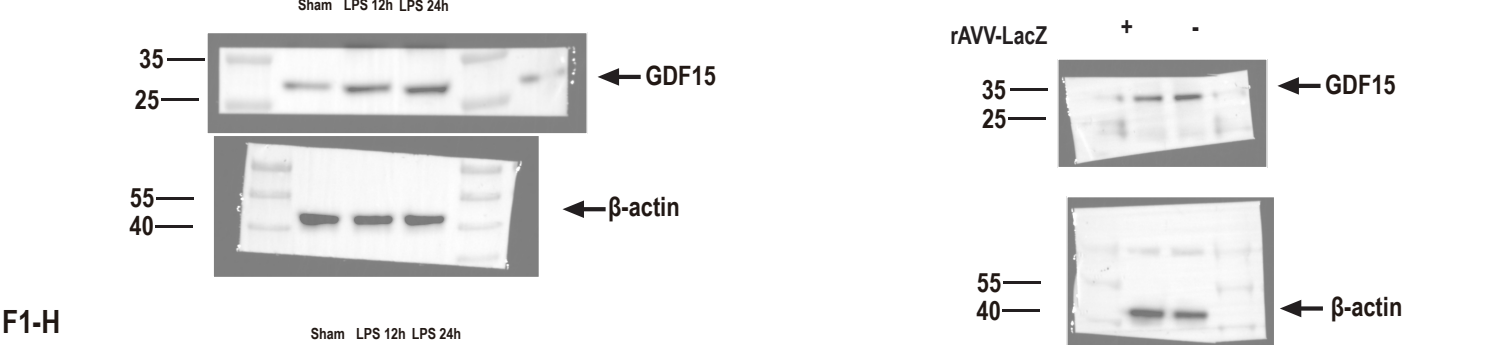

F1-H

F2-H

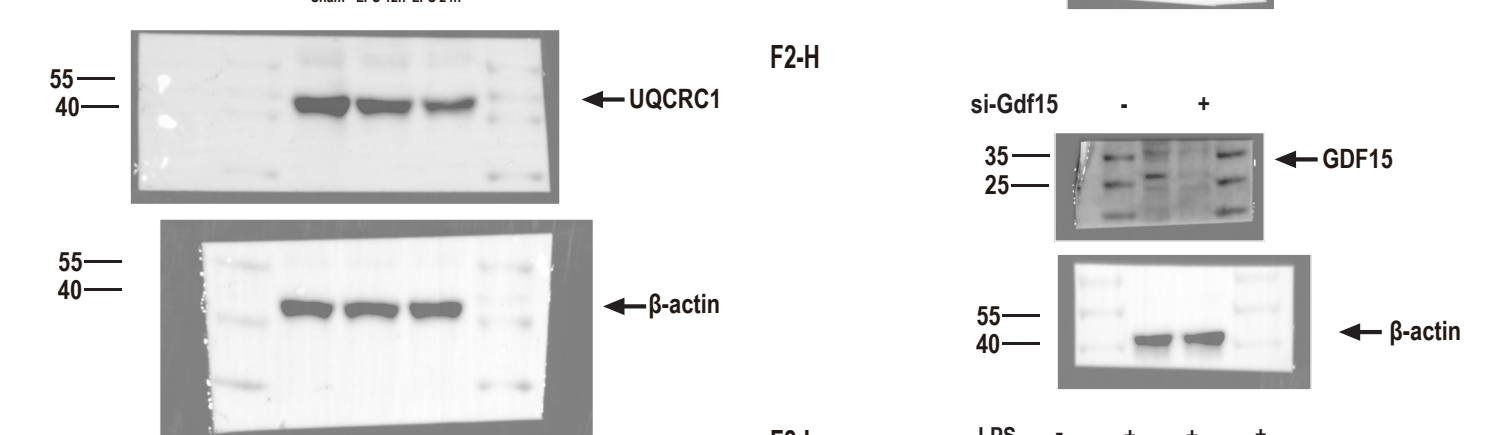

F1-I

F2-I

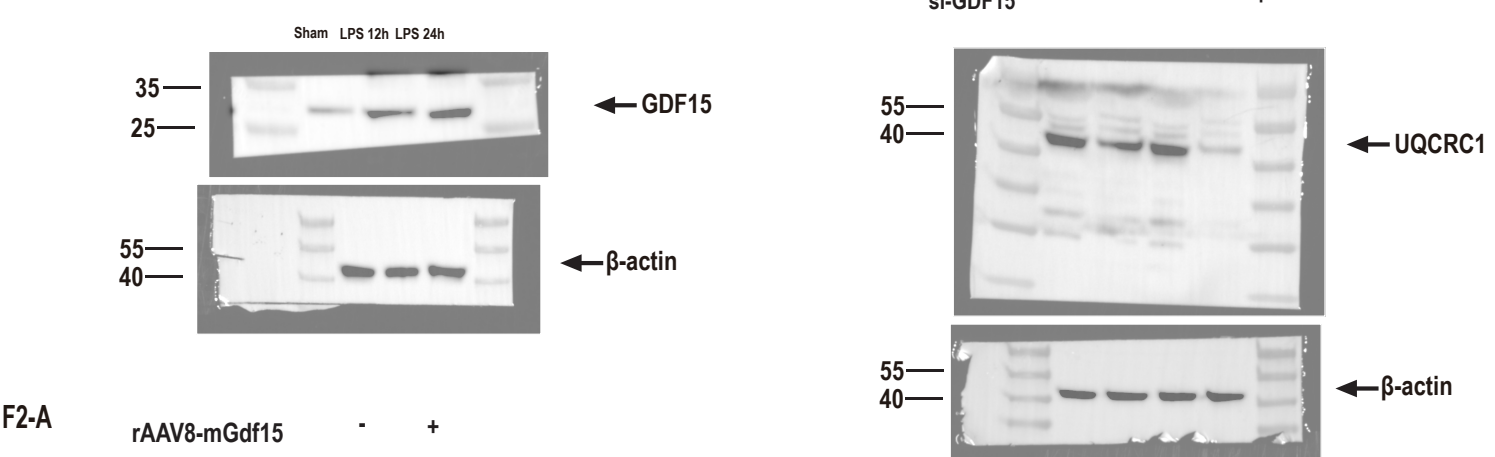

F2-A

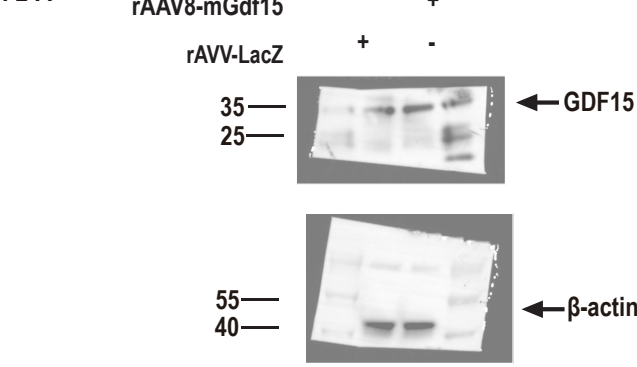

Supplement: Supplementary file 2 [file DataSheet1.pdf]

F3-A

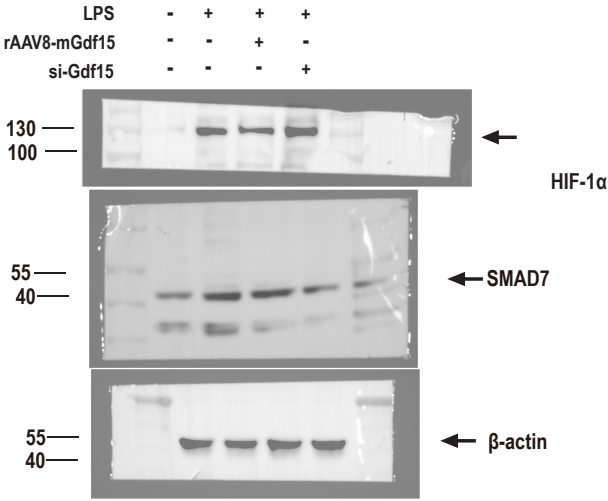

F3-B

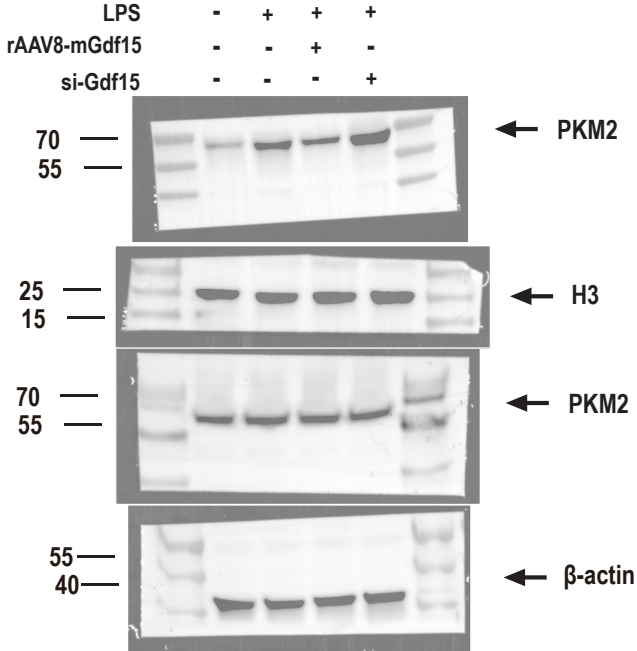

F4-A

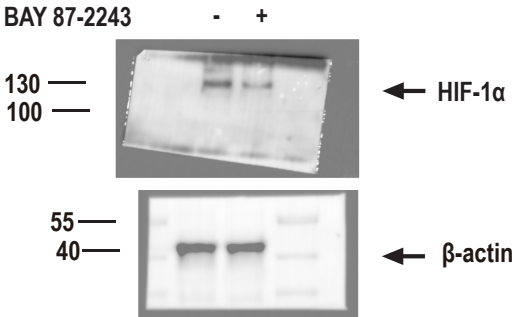

F4-B

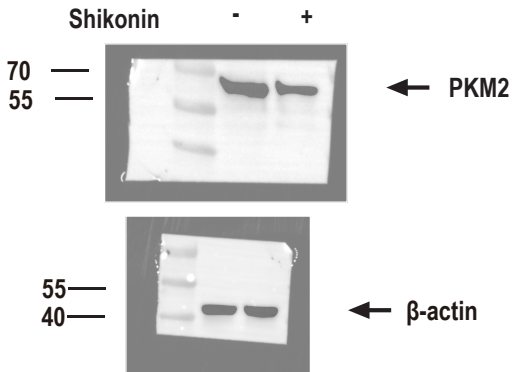

F4-C

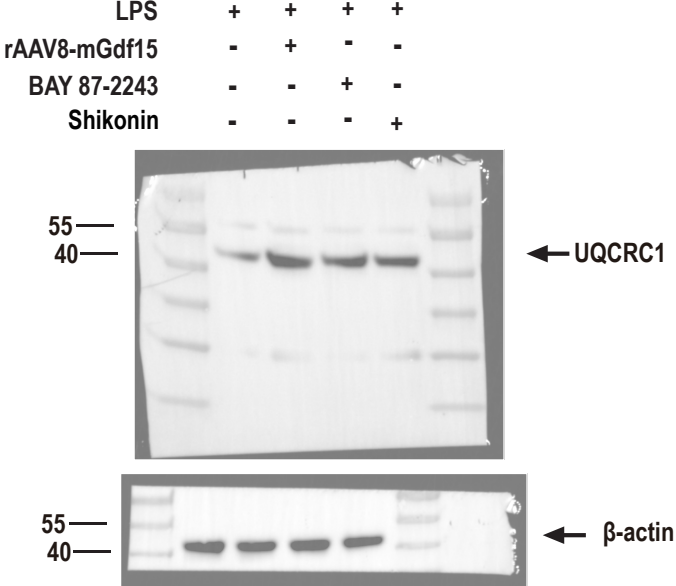

F4-E

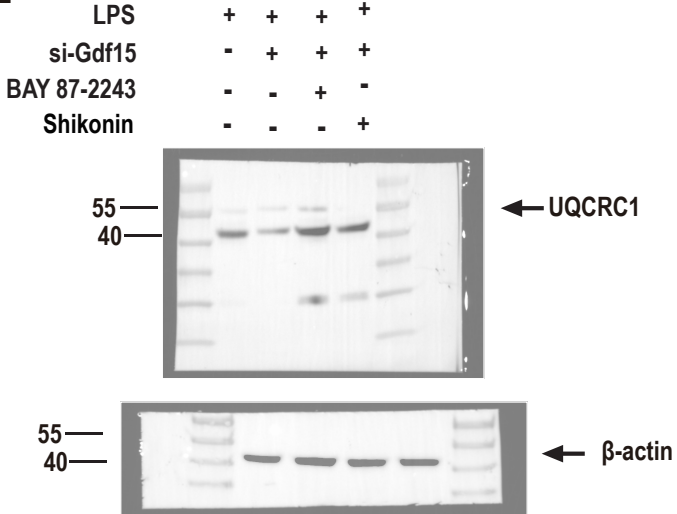

F5-A

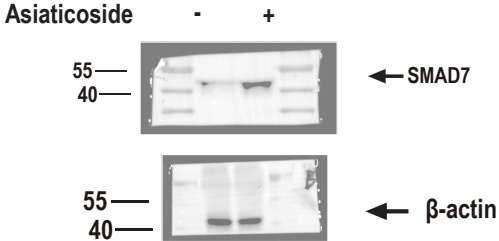

F5-B

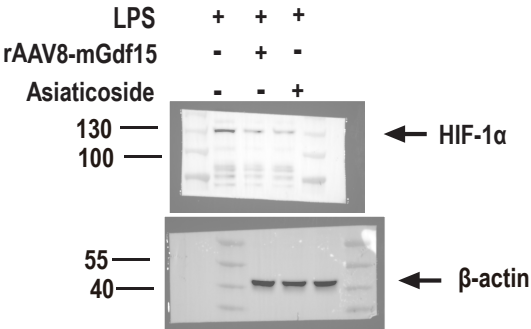

F5-C

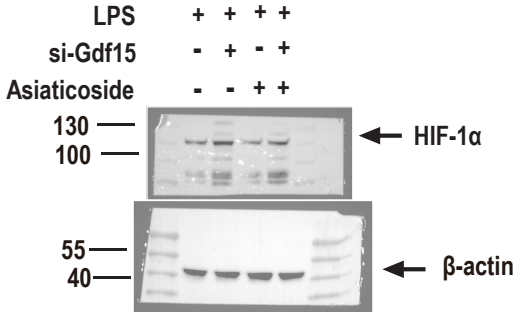

Supplement: Supplementary file 3 [file DataSheet2.pdf]
